# Cerebral response to subject’s own name showed high prognostic value in traumatic vegetative state

**DOI:** 10.1186/s12916-015-0330-7

**Published:** 2015-04-15

**Authors:** Fuyan Wang, Haibo Di, Xiaohua Hu, Shan Jing, Aurore Thibaut, Carol Di Perri, Wangshan Huang, Yunzhi Nie, Caroline Schnakers, Steven Laureys

**Affiliations:** International Vegetative State and Consciousness Science Institute, Hangzhou Normal University, Hangzhou, China; Department of Radiology, The Affiliated Hospital of Hangzhou Normal University, Hangzhou, China; Department of Rehabilitation, Hangzhou Wujing Hospital, Hangzhou, China; Coma Science Group, GIGA-Research, University and University Hospital of Liège, Liège, Belgium; Department of Neurosurgery, University of California, Los Angeles, CA USA

**Keywords:** Functional MRI, Own name, Prognosis, Traumatic brain injury, Vegetative state/unresponsive wakefulness syndrome

## Abstract

**Background:**

Previous studies have shown the prognostic value of stimulation elicited blood-oxygen-level-dependent (BOLD) signal in traumatic patients in vegetative state/unresponsive wakefulness syndrome (VS/UWS). However, to the best of our knowledge, no studies have focused on the relevance of etiology and level of consciousness in patients with disorders of consciousness (DOC) when explaining the relationship between BOLD signal and both outcome and signal variability. We herein propose a study in a large sample of traumatic and non-traumatic DOC patients in order to ascertain the relevance of etiology and level of consciousness in the variability and prognostic value of a stimulation-elicited BOLD signal.

**Methods:**

66 patients were included, and the response of each subject to his/her own name said by a familiar voice (SON-FV) was recorded using fMRI; 13 patients were scanned twice in the same day, respecting the exact same conditions in both cases. A behavioral follow-up program was carried out at 3, 6, and 12 months after scanning.

**Results:**

Of the 39 VS/UWS patients, 12 (75%) out of 16 patients with higher level activation patterns recovered to minimally conscious state (MCS) or emergence from MCS (EMCS) and 17 (74%) out of 23 patients with lower level activation patterns or no activation had a negative outcome. Taking etiology into account for VS/UWS patients, a higher positive predictive value was assigned to traumatic patients, i.e., up to 92% (12/13) patients with higher level activation pattern achieved good recovery whereas 11 out of 13 (85%) non-traumatic patients with lower level activation or without activation had a negative clinical outcome. The reported data from visual analysis of fMRI activation patterns were corroborated using ROC curve analysis, which supported the correlation between auditory cortex activation volume and VS/UWS patients’ recovery. The average brain activity overlap in primary and secondary auditory cortices in patients scanned twice was 52%.

**Conclusions:**

The activation type and volume in auditory cortex elicited by SON-FV significantly correlated with VS/UWS patients’ prognosis, particularly in patients with traumatic etiology, however, this could not be established in MCS patients. Repeated use of this simple fMRI task might help obtain more reliable prognostic information.

## Background

The prognostic value of blood-oxygen-level-dependent (BOLD) signals elicited by various sensory stimuli in vegetative state/unresponsive wakefulness syndrome [1,2] (VS/UWS) patients has been shown in several studies [3]. However, the variability of stimulation paradigms among these studies may have limited the deductive power for the prognostic value of the BOLD signals in DOC patients. Furthermore, none of them mentioned the role of etiology.

It is known that the etiology of brain injury affects patients’ recovery. Traumatic brain injuries are associated with better outcomes at one year than non-traumatic injuries [4,5], suggesting that the prognostic value of BOLD signals in this challenging population of patients should be explored for traumatic and non-traumatic patients separately.

Our previous work showed the prognostic value of a BOLD signal elicited by patients’ own name spoken by a familiar voice (SON-FV) in two patients diagnosed as traumatic VS/UWS [6].

As demonstrated in the ‘cocktail party’ phenomenon, a person’s own name is the most powerful, emotionally laden auditory stimuli to gain entry to awareness [7]. It has been reported that the subject’s own name (SON) activated the cerebral cortex more extensively versus non-self-referential emotional stimuli in patients with MCS [8] and the SON spoken by a familiar voice (SON-FV), versus an unfamiliar voice, elicited stronger event-related potential responses [9]. Furthermore, we recently found that the SON showed higher sensitivity to elicit sound localization reflex in DOC patients [10]. Given these findings, we chose to present the SON-FV to maximize our chances of detecting residual brain function in a larger number of DOC patients using functional MRI (fMRI), and verify its prognostic value in clinical daily use. Taking into account DOC patients’ fluctuating level of consciousness [11], we also decided to test the SON-FV brain activation consistency in a sub-population of patients scanned twice.

## Methods

### Participants

Overall, 74 DOC patients with severe brain injury were included in this study. After fMRI scanning, 8 were excluded due to head movement parameters (after scanning, patients’ head movement parameters were extracted and excluded from the threshold list as: translation >8 mm or roll angle >2°). Of the remaining 66 patients (52 male, 14 female, age range 2 to 73 years, mean 39 years; etiology: 43 traumatic, 12 anoxic brain injury, 10 cerebrovascular accident, 1 meningitis; time between ictus and fMRI scanning: 1 to 60 months, mean 8.5 months), 39 patients met the diagnostic criteria defining VS/UWS (23 traumatic and 16 non-traumatic), 25 patients met the diagnostic criteria defining MCS (19 traumatic and 6 non-traumatic), and 2 patients were diagnosed as EMCS (1 traumatic and 1 non-traumatic) according to the Coma Recovery Scale-Revised (CRS-R) [12] (see Table 1 for detailed demographic and clinical information). Data on 11 of the 66 patients (VS/UWS: 24, 25, 26, 27, 28, 29, and 30; MCS: 14, 15, 16, and 17) has previously been reported [6].

**Table 1:** **The characteristic data of 66 patients in DOC**

**Pats.**	**Diag.**	**Sex/age, y**	**Cause**	**Lesions (CT or MRI)**	**Mon. after insult**	**Left auditory cortex(mm** ^**3**^ **)**	**Right auditory cortex(mm** ^**3**^ **)**	**3 mon. diagnosis**	**6 mon. diagnosis**	**12 mon. diagnosis**	**Activation type**
VS1	VS	M/47	TBI	Brain stem lesions	8	0	0	VS	VS	VS	No
VS2	VS	F/55	CVA	Right temporal lobe lesions	26	14,025 (165)	13,005 (153)	VS	VS	VS	High
VS3	VS	M/20	TBI	Left temporal lobe lesions	20	0	10,115 (119)	VS	VS	MCS	High
VS4	VS	M/3Y.2 M	Anoxic brain injury	Diffuse brain contusion	4	1,700 (20)	0	VS	VS	VS	Primary
VS5	VS	M/64	TBI	Bilateral frontal and left temporal lobe lesions	8	8,245 (97)	0	VS	MCS	MCS	High
VS6	VS	M/59	TBI	Bilateral fontal, left parietal, and temporal lobe lesions	1	0	15,725 (185)	EMCS	MCS	MCS	High
VS7	VS	M/73	CVA	Right cerebellum lesions	2	2,210 (26)	3,485 (41)	MCS	VS	VS	Primary
VS8	VS	F/3	Meningitis	—	5	0	4,250 (50)	VS	VS	Died	Primary
VS9	VS	M/47	Anoxic brain injury	—	2	2,125 (25)	0	MCS	EMCS	EMCS	Primary
VS10	VS	F/69	TBI	Bilateral frontal, parietal lobe, and left temporal lobe lesions	4	15,130 (178)	0	VS	VS	VS	High
VS11	VS	M/20	TBI	Diffuse brain lesion	24	12,325 (145)	0	MCS	MCS	MCS	High
VS12	VS	M/31	TBI	Brain stem, right temporal lobe, and left frontal lobe lesions	6	0	1,275 (15)	MCS	MCS	MCS	Primary
VS13	VS	M/48	TBI	Right frontal, temporal, parietal, and left frontal lobe lesions	2	30,515 (150)	6,375 (75)	MCS	Died	Died	High
VS14	VS	M/54	CVA	Right frontal and temporal lobe lesions	2	0	1,360 (16)	VS	VS	Died	Primary
VS15	VS	M/27	TBI	Diffuse brain contusion, subarachnoid hemorrhage	1	0	0	EMCS	EMCS	EMCS	No
VS16	VS	M/28	TBI	Diffuse brain hemorrhage	60	0	0	MCS	MCS	—	No
VS17	VS	M/17	Anoxic brain injury	Diffuse cortical atrophy	6	0	5,355 (63)	VS	VS	VS	High
VS18	VS	F/31	TBI	Right frontal and temporal lobe lesions	2	24,650 (290)	9,265 (109)	MCS	MCS	MCS	High
VS19	VS	F/8	TBI	Subdural fluid accumulation, cerebromalacia in right basal ganglia	3	0	23,630 (278)	MCS	MCS	MCS	High
VS20	VS	M/36	Anoxic brain injury	Diffuse brain contusion	2	0	2,550 (30)	VS	VS	VS	Primary
VS21	VS	M/24	Anoxic brain injury	Subarachnoid hemorrhage, diffuse brain contusion	2	1,360 (16)	2,380 (28)	VS	—	—	Primary
VS22	VS	M/60	TBI	Left parietal lobe hemorrhage	3	20,995 (247)	0	MCS	MCS	MCS	High
VS23	VS	M/19monh	TBI	Extensive brain lesions	2	9,010 (106)	40,885 (481)	MCS	MCS	MCS	High
VS24	VS	M/29	Anoxic brain injury	Diffuse cortical atrophy	48	1,360 (16)	0	VS	VS	VS	Primary
VS25	VS	M/42	TBI	Diffuse brain lesions	2	0	1,275 (15)	VS	VS	—	Primary
VS26	VS	F/52	TBI	Right frontal and left temporal lobe lesions	2	0	765 (9)	VS	VS	—	Primary
VS27	VS	M/38	TBI	Left occipital lobe and bilateral basal ganglia lesions	4	20,995 (247)	8,330 (98)	MCS	MCS	—	High
VS28	VS	M/21	TBI	Right temporal, frontal, and parietal lobe lesions	4	15,385 (180)	4,505 (53)	MCS	MCS	—	High
VS29	VS	M/58	Anoxic brain injury	Temporal and parietal lobe lesions	4	0	0	VS	VS	—	No
VS30	VS	M/61	TBI	Left temporal and frontal lobe lesions	8	0	0	VS	VS	Died	No
VS31	VS	M/63	TBI	Frontal and right temporal lobe contusion	18	0	2,530 (22)	VS	MCS	MCS	Primary
VS32	VS	F/45	TBI	Left subdural hematoma and extensive brain contusion	3	3,105 (27)	4,370 (38)	MCS	MCS	MCS	High
VS33	VS	M/45	TBI	Left epidural hematoma	12	3,450 (30)	0	VS	VS	VS	Primary
VS34	VS	M/23	Anoxic brain injury	Diffuse cortical atrophy	17	0	2,645 (23)	VS	VS	VS	Primary
VS35	VS	M/20	Anoxic brain injury	Diffuse cortical atrophy	7	0	0	VS	VS	VS	No
VS36	VS	M/43	CVA	Bilateral basal ganglia hemorrhage	3	19,090 (166)	8,970 (78)	VS	VS	VS	High
VS37	VS	M/21	TBI	Diffuse axonal injury, subarachnoid hemorrhage	6	3,680 (32)	0	VS	VS	VS	Primary
VS38	VS	F/38	Anoxic brain injury	Lateral ventricle expansion	3	0	0	VS	VS	VS	No
VS39	VS	M/54	Anoxic brain injury	Diffuse cortical atrophy	2	0	2,760 (24)	VS	VS	—	Primary
MCS1	MCS	F/46	TBI	Left temporal lobe lesions	3	0	11,645 (137)	MCS	EMCS	EMCS	High
MCS2	MCS	M/42	TBI	Bilateral frontal and left temporal lobe lesions	5	4,165 (49)	0	MCS	EMCS	EMCS	High
MCS3	MCS	M/45	TBI	Diffuse brain lesions	7	50,830 (598)	27,880 (328)	MCS	MCS	—	High
MCS4	MCS	M/46	TBI	Right frontal, temporal, and parietal lobe hematoma	4	12,580 (148)	5,100 (60)	VS	VS	VS	High
MCS5	MCS	M/33	TBI	Bilateral frontal and left parietal lobe lesions	1	12,750 (150)	0	EMCS	EMCS	EMCS	High
MCS6	MCS	F/29	TBI	Left frontal and parietal lobe hematoma	2	1,020 (12)	0	EMCS	EMCS	EMCS	Primary
MCS7	MCS	M/21	TBI	Left temporal hematoma and right subdural hematoma	3	9,095 (107)	28,390 (334)	MCS	MCS	Died	High
MCS8	MCS	M/65	TBI	Bilateral frontal lobe lesions, subarachnoid hemorrhage	3	0	19,550 (230)	EMCS	EMCS	EMCS	High
MCS9	MCS	M/20	TBI	Subarachnoid hemorrhage	3	23,120 (272)	12,240 (144)	MCS	MCS	MCS	High
MCS10	MCS	M/19	TBI	Diffuse axonal contusion	1	3,655 (43)	4,845 (57)	EMCS	EMCS	EMCS	High
MCS11	MCS	M/60	TBI	Left frontal and temporal hematoma	2	0	10,710 (126)	MCS	MCS	—	High
MCS12	MCS	M/32	TBI	Right frontal, bilateral temporal and parietal lobe lesions	2	935 (11)	1,445 (17)	EMCS	EMCS	EMCS	Primary
MCS13	MCS	F/64	CVA	Subarachnoid hemorrhage	8	7,140 (84)	0	EMCS	EMCS	EMCS	High
MCS14	MCS	M/30	TBI	Right temporal and frontal lobe lesions	2	23,120 (272)	16,405 (193)	MCS	EMCS	—	High
MCS15	MCS	F/24	TBI	Subdural hematoma, brainstem lesions	3	25,160 (296)	15,470 (179)	MCS	EMCS	—	High
MCS16	MCS	M/38	TBI	Bilateral temporal and frontal lobe lesions	6	0	22,780 (268)	MCS	MCS	—	High
MCS17	MCS	M/30	TBI	Left temporal and bilateral frontal lobe lesions	26	0	18,275 (215)	MCS	MCS	—	High
MCS18	MCS	M/50	CVA	Left basal ganglia and brain stem hemorrhage	14	0	805 (7)	MCS	MCS	MCS	Primary
MCS19	MCS	F/56	CVA	Brian stem hemorrhage	24	0	3,335 (29)	MCS	MCS	MCS	High
MCS20	MCS	F/59	CVA	Left basal ganglia and paraventricle hemorrhage	5	0	3,995 (47)	MCS	MCS	MCS	High
MCS21	MCS	M/62	CVA	Left cerebellum hemorrhage	17	1,035 (9)	0	MCS	MCS	MCS	Primary
MCS22	MCS	M/37	TBI	Bilateral frontal and left basal ganglia hemorrhage	4	0	2,760 (24)	EMCS	EMCS	EMCS	Primary
MCS23	MCS	M/42	TBI	Bilateral frontal and temporal contusion and hemorrhage	24	7,990 (94)	0	EMCS	EMCS	EMCS	High
MCS24	MCS	M/50	TBI	Brain stem, left temporal, right frontal and bilateral occipital contusion	9	8,925 (105)	0	MCS	MCS	MCS	High
MCS25	MCS	M/23	Anoxic brain injury	Diffuse axonal atrophy	28	0	11,845 (103)	MCS	MCS	MCS	High
EMCS1	EMCS	M/32	CVA	Bilateral basal ganglia, brain stem, and right cerebellum lesions	13	0	3,740 (44)	EMCS	EMCS	EMCS	Primary
EMCS2	EMCS	M/58	TBI	Left subdural hematoma	2	11,560 (136)	9,690 (114)	EMCS	EMCS	EMCS	High

Show the characteristic data, activation volume (mm^3^) and voxel number (in brackets) of right and left side of auditory cortex, the follow-up diagnosis at 3, 6, and 12 months, and the activation type (Low, No, Lower level; High, higher level) of the patients with disorders of consciousness. TBI, Traumatic brain injury; CVA, Cerebrovascular accident; MCS, Minimally conscious state; EMCS, Emergence from minimally conscious state; VS, Vegetative state. Consent for the publication of the information relating to individual participants was obtained from the legal representative of all participants.

All patients were admitted to the rehabilitation units of Hangzhou Wujing hospital and Taizhou municipal hospital. Prior to admission, all patients were verified suitable for MRI scanning by a team of expert neurologists. Patients who had suffered brain injury less than one month before the time of evaluation were excluded from the study. An observation of patients’ baseline head movement was also performed at bedside by caregivers to check the patients were able to stay in the scanner for at least 10 minutes without excessive head movement. Informed written consent was obtained from the physician and family of each patient. Written informed consent for the publication of patient details was also obtained from the legal representative of all patients. The study was approved by the Ethics Committee of Hangzhou Normal University School of Basic Medicine.

Fifteen volunteer college undergraduate students (9 female, age range 18 to 27 years, mean age 24 years) participated in the study as healthy controls. All the volunteers had normal hearing and normal or corrected-to-normal vision. None reported any history of head injury or neurological or psychiatric disorders. Written informed consent was obtained from each participant prior to the experiment according to a protocol approved by the Ethics Committee of Hangzhou Normal University School of Basic Medicine.

### Behavioral assessment

All patients recruited to the study underwent behavioral assessment employing the CRS-R before fMRI data acquisition. In order to obtain a significant prognostic value, a follow-up program was conducted at 3, 6, and 12 months after fMRI data acquisition. If the patients were discharged from or transferred to other hospitals during this tracking program, a phone call follow-up was performed. The assessors for determining outcome were blinded for the fMRI results.

### Image data acquisition and analysis

Before acquiring neuroimaging data, we digitally recorded and adapted the SON-FV using the voice of a first-degree relative and GoldWave software (GoldWave Inc.). fMRI scanning was performed using block design with six active blocks and seven baseline blocks for each patient. Each active block lasted 12 seconds and included seven SON-FVs (each name lasted 1 sec). Each baseline block consisted of 18 seconds of attenuated machine noise; 13 patients were scanned twice in the same day (MRI scanner and conditions were the same in both cases) to obtain the overlap rate of two separate scans with the same fMRI paradigm. The auditory stimuli were presented through MRI-compatible noise-attenuated headphones (Resonance Technology, Inc., Los Angeles, CA) binaurally. A special designed head-fixation devise (a pumping pillow) and polystyrene foam was used for every patient to reduce spontaneous head movement. Data were acquired using a 1.5 T General Electrics Sigma Horizon MRI system and a 1.5 T Siemens Magnetom Essenza MRI system (15 controls, VS/UWS1-30 and MCS1-17using GE MRI; VS/UWS31-39, MCS17-25, and EMCS1-2 using Siemens MRI). When scanning, first, 22 axial anatomic images were collected using a T1-weighted spin echo sequence (repetition time = 500 msec, echo time = 9 msec, field of view = 240 × 240 mm, slice thickness = 5 mm, skip = 1 mm, matrix = 256 × 256, with the resolution of three dimensions of one voxel: x = 0.9375 mm, y = 0.9375 mm, z = 6 mm). Next, 96 (144) images per slice were acquired using a gradient echo planar imaging (repetition time = 3,000 or 2,000 msec, matrix = 64 × 64, with the resolution of three dimensions of one voxel: x = 3.75 mm, y = 3.75 mm, z = 6 mm). Finally, a fast spoiled gradient recalled sequence (repetition time = 27 msec, echo time = 6 msec, field of view = 240 × 240 mm, matrix = 256 × 256, with the resolution of three dimensions of one voxel: x = 1.3 mm, y = 0.9375 mm, z = 0.9375 mm) was used in a sagittal plane to collect three-dimensional images covering the entire volume of the brain. The imaging procedures and parameters were similar to those of our previously published studies [6].

Analysis of Functional NeuroImages (AFNI) software (version release in later 2009) was used for data analysis [13]. After correcting for two- and three-dimensional head motion, the functional images were smoothed using an isotropic Gaussian kernel (full width at half maximum = 6 mm). We then used multiple linear regression analysis (using the 3Ddeconvolve program in AFNI) to further correct the head movement artifacts (six estimated motion-induced time series used as non-interest regressors). Finally, a first level fixed-effect statistics was performed using general linear model for each patient at whole-brain level to identify SON-FV-induced BOLD signal increases for generating activation maps.

Due to the inconsistency of spontaneous head movements, we selected a statistical threshold of *t* >2 (*P* <0.05, corrected). To avoid false-negative results, a minimum cluster size of 10 voxels was used as an extent threshold. For 13 twice-scanned patients, we separately proceeded data analysis, and then chose the scan with better activation (more volumes or higher *t* value) as the final result for statistical analysis in the next step.

Since it can be difficult to accurately identify these cortical areas in deformed brains, we deemed normalized analysis to be unsuitable for this cohort of patients with severe brain injury. Thus, after fitting all the patients activation maps individually to their respective structural MRI data, the Heschl gyrus (HG) was defined as the primary auditory cortex [14,15] (if two HG were present, the anterior gyrus was termed area 41 and the posterior gyrus area 42), whilst the planum temporale, the planum polare [16], and the posterior and lateral extensions of HG were defined as the auditory cortices termed area 21/22. Based on these definitions, we chose the bilateral auditory associate cortex in the temporal area as region of interest in each patient after the anatomic landmarks in three orthogonal cross-sectional views [17,18] (axial, coronal, and sagittal) of the individual high-resolution three-dimensional brain images were repeatedly and simultaneously checked by an experienced radiologist (all these steps were carried out using AFNI plugins). Whether the activation of each patient extended to a higher order auditory cortex or not was also determined.

In the case of most VS/UWS patients there was either no activation or activation was found in the primary auditory cortex, which is defined as ‘lower level’ activation. The activation in some VS/UWS patients may extend to a higher order associative auditory cortex (e.g., area 21/22), similar to the activation pattern observed in MCS patients or healthy controls; this type was defined as ‘higher level’ activation [3].

Finally, Receiver Operating Characteristic (ROC) curve analysis was chosen to analyze the prognostic value of primary and secondary auditory cortex (bilateral) activation volume in VS/UWS and MCS patients [19]. Fisher’s exact test looked for differences in outcome depending on the type of cerebral activation. Results were considered significant at *P* <0.05.

## Results

### Activation

**Figure 1:**
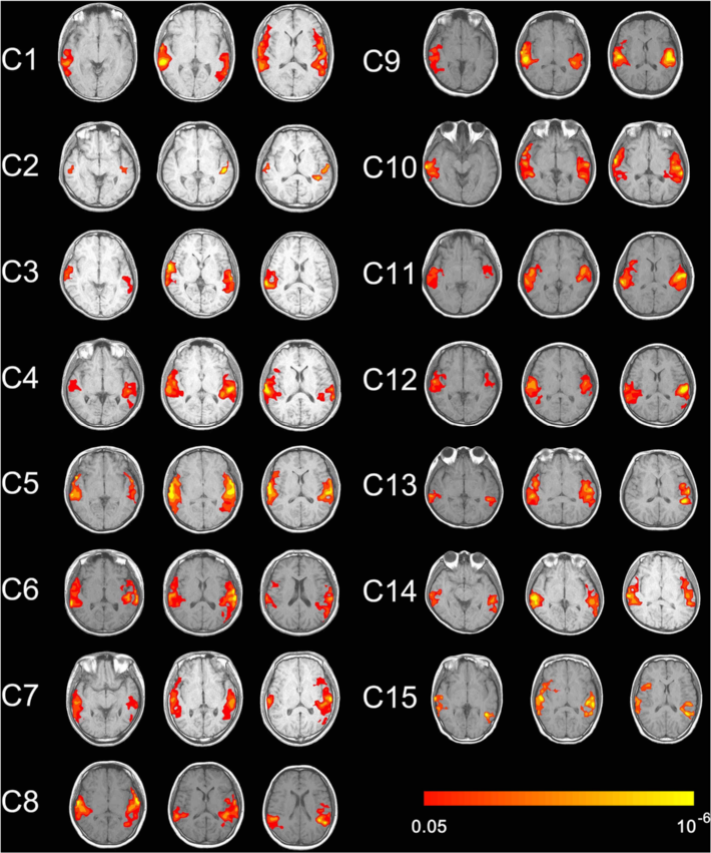
Show activation of auditory cortex caused by own name stimulation in 15 controls (axis view, P <0.05, corrected).

In 15 healthy controls, each participant had significant activation not only in primary auditory cortices but also extending to higher order associative auditory cortices (higher level activation, *P* <0.05, corrected) (Figure 1).

**Figure 2:**
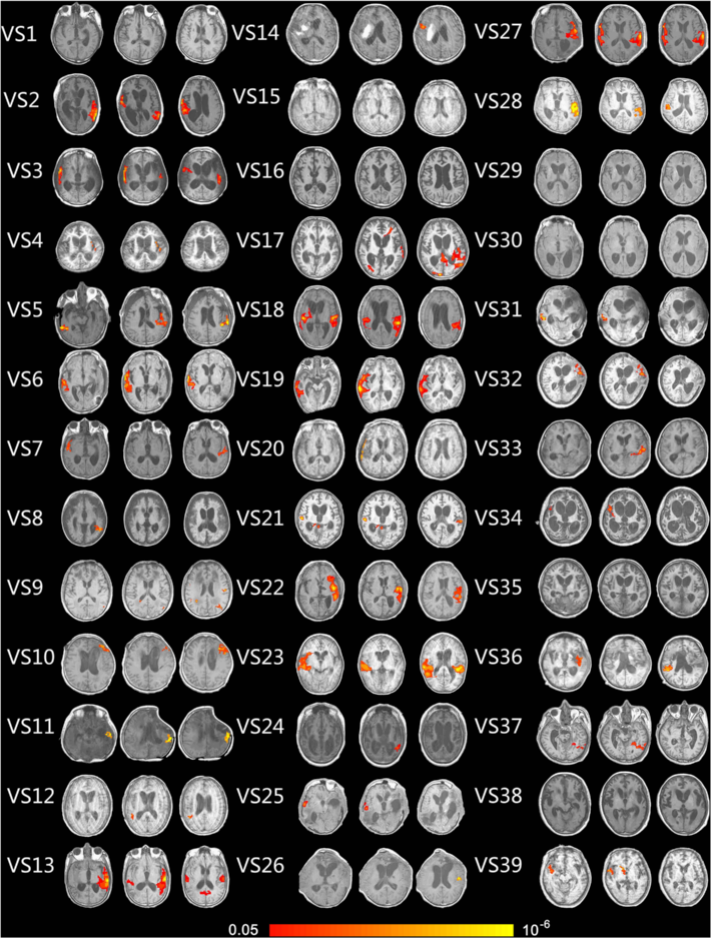
Show activation of auditory cortex caused by own name stimulation in 39 VS/UWS patients (axis view, P <0.05, corrected).

**Figure 3:**
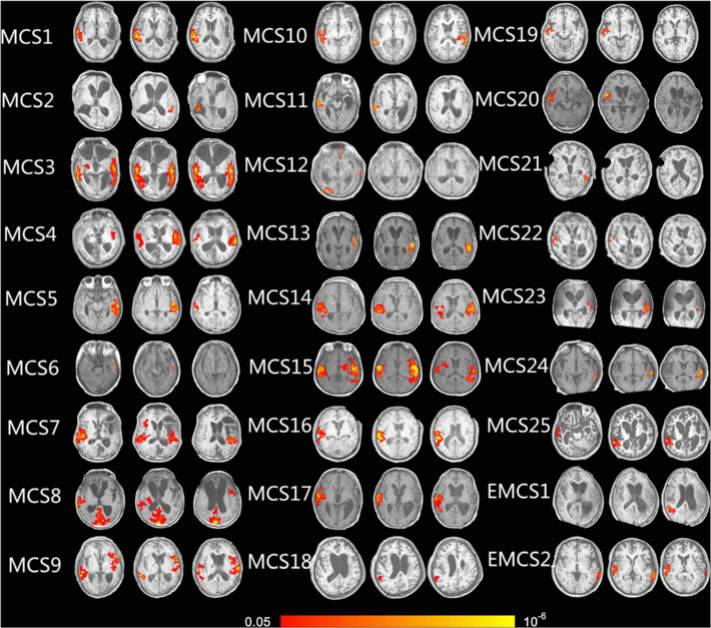
Show activation of auditory cortex caused by own name stimulation in 25 MCS and 2 EMCS patients (axis view, P <0.05, corrected).

In 39 VS/UWS patients, 7 patients had no activation at all in the auditory cortex (VS/UWS: 1, 15, 16, 29, 30, 35, and 38; 5 traumatic and 2 non-traumatic) and 16 patients had significant activation in the primary auditory cortex (VS/UWS: 4, 7, 8, 9, 12, 14, 20, 21, 24, 25, 26, 31, 33, 34, 37, and 39; 6 traumatic and 10 non-traumatic). A more extensive activation encompassing HG and area 21/22 was observed in 16 patients (VS/UWS: 2, 3, 5, 6, 10, 11, 13, 17, 18, 19, 22, 23, 27, 28, 32, and 36; 13 traumatic and 3 non-traumatic). In 25 MCS patients, 5 had activation in primary auditory cortices (MCS: 6, 12, 18, 21, 22; 2 traumatic and 3 non-traumatic) and the others had activation in primary auditory cortices which extended to higher order associative auditory cortices (MCS1–5: 7–11, 19, 20, 23–25; 16 traumatic and 4 non-traumatic). In 2 EMCS patients, 1 had significant activation in primary auditory cortices extending to higher order associative auditory cortices (EMCS 2, traumatic), the other’s activation was limited to the primary auditory cortex (EMCS 1, non-traumatic) (Figures 2 and 3).

### Prognosis

Using the 12-month behavioral follow-up data (based on CRS-R score) of the 39 VS/UWS patients for prognostic value statistics, 12 out of 16 (75%) VS/UWS patients (13 traumatic, 3 non-traumatic) with higher level activation (extending to higher order associate auditory cortex) recovered to MCS or EMCS (this kind of recovery was taken as a good outcome), whereas 17 (74%) out of 23 VS/UWS patients (10 traumatic, 13 non-traumatic) with no activation or activation limited to the primary auditory cortex had a poor outcome (remaining in VS/UWS). The sensitivity and specificity of this method for VS/UWS patients was 67% and 81%, respectively (Tables 2 and 3). Outcome differed depending on activation type (*P* = 0.004). Of the 5 MCS patients with activation in only the primary auditory cortex, 3 recovered to EMCS (3 traumatic) and 2 were still diagnosed as MCS (2 non-traumatic). Of the 20 MCS patients with activation in a higher order associate auditory cortex, 9 recovered to EMCS (8 traumatic and 1 non-traumatic), 10 remained in MCS (7 traumatic and 3 non-traumatic), and 1 patient (traumatic) was unexpectedly diagnosed as VS/UWS; 2 EMCS patients achieved no behavioral recovery.

**Table 2:** **Prognostic value of activation type in VS/UWS patients**

**Cerebral activation**	**No activation or primary auditory cortex activation**	**Higher level activation beyond primary auditory cortex**	**Total**
Bad outcome	17	4	21
Good outcome	6	12	18
Total	23	16	39

Showed predictive value (sensitivity = 66.7%, specificity = 81.0%) of activation pattern (lower and higher level) in VS/UWS patients; 12 out of 16 (75%) VS/UWS patients with higher level activation recovered to MCS or EMCS, whereas 17 (73.9%) out of 23 VS/UWS patients with no activation or activation limited to the primary auditory cortex had a bad outcome (remaining in VS/UWS). Outcome differed depending on activation type (*P* = 0.004, Fisher’s exact testing).

**Table 3:** **Prognostic value of activation type in traumatic or non-traumatic VS/UWS patients**

**1. Traumatic**			
**Activation**	**No activation or primary auditory cortex activation**	**Higher level activation beyond primary auditory cortex**	**Total**
Etiology	Traumatic brain injury	Traumatic brain injury	
Good Outcome	4	12	16
Bad Outcome	6	1	7
Total	10	13	23
**2. Non-traumatic**			
**Activation**	**No activation or primary auditory cortex activation**	**Higher level activation beyond primary auditory cortex**	**Total**
Etiology	Non-traumatic brain injury	Non-traumatic brain injury	
Good Outcome	2	0	2
Bad Outcome	11	3	14
Total	13	3	16

In 23 traumatic VS/UWS patients, 12 (92.3%) out of 13 patients with higher level activation beyond primary auditory cortex had a good recovery, whereas 6 (60%) out of 10 traumatic VS/UWS patients with no activation or primary auditory cortex activation had a bad outcome. Outcome differed depending on activation type (*P* = 0.019, Fisher’s exact testing).

In 16 non-traumatic VS/UWS patients, 11 (85%) out 13 patients with no or primary auditory cortex activation had a bad outcome. None of 3 patients with higher level activation beyond primary auditory cortex had a good recovery.

The 39 VS/UWS patients were divided into two groups according to etiology; traumatic (n = 23) and non-traumatic (n = 16) patients. Of the 23 traumatic VS/UWS patients, 12 (92%) out of 13 with higher level activation pattern had a good outcome (i.e., recovered to MCS or EMCS, *P* = 0.019). Of the 16 non-traumatic VS/UWS patients, 11 (85%) out of 13 with no or only primary auditory cortex activation had a bad outcome (Table 3).

Of the 25 MCS patients, 9 out of the 12 patients who had recovered at the following behavioral assessment had activation beyond the primary cortex and, hence, the sensitivity of higher level activation in MCS patients was 75%. Only 2 of the 13 patients with a poor outcome had lower level activation (specificity = 15%).

Considering etiology, in the MCS traumatic group (n = 19), 8 out of 16 (50%) patients with higher level activation achieved good recovery. All (3/3) patients with primary lower level activation had a poor outcome. Of the 6 non-traumatic MCS patients, 1 out of 4 (25%) patients with higher order cortex activation recovered. All 2 (100%) patients with only primary cortex activation had a poor outcome.

Analysis of the BOLD signal prognostic value also considered patient age. With patient age <40, 10 out of the 11 traumatic VS/UWS patients achieved good recovery, independently of higher level activation. Only 6 out of the 12 traumatic VS/UWS patients >40 years old showed good recovery. The only traumatic VS/UWS patient with a higher level activation pattern and a bad outcome was 69 years old.

Using a ROC curve, we correlated activation volume with the prognostic outcome, as indicated by follow-up CRS-R scores. In all 39 VS/UWS patients, the activation volume of bilateral primary and secondary auditory cortices significantly correlated to a positive prognostic outcome when >5.355 cm^3^ (Sensitivity = 72%, Specificity = 86%, *P* <0.0039). In 23 traumatic VS/UWS patients, the activation volume of bilateral primary and secondary auditory cortices significantly correlated with the recovery when >3.680 cm^3^ (Sensitivity = 75%, Specificity = 86%, *P* <0.0015). No statistically significant findings were obtained for MCS patients, not even when patient etiology was taken into account. Two EMCS patients were not included in the statistical analysis.

### Overlap in 13 twice-scanned patients

**Figure 4:**
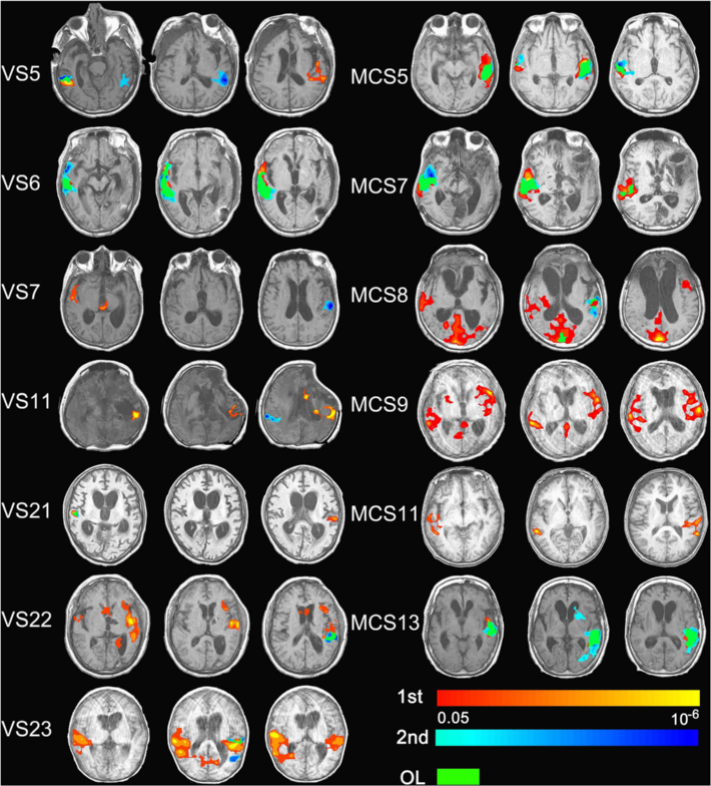
Show activation in auditory cortex of the 13 patients scanned twice on the same day (7 VS/UWS and 6 MCS) under the exact same acquiring conditions. Red, activation of first own name stimulation; blue, activation of second own name stimulation; Green, overlap area between two scans; axis view, *P* <0.05, corrected).

Of the 13 patients scanned twice on the same day (7 VS/UWS and 6 MCS) under the exact same acquiring conditions, 1 MCS patient had a brain activity overlap of 100% in both scans, 8 (5 VS/UWS, 3 MCS) out of 13 patients had a 50% or 75% overlap in the two separate scans, 2 (1 VS/UWS, 1 MCS) patients had an overlap of 25%, and 2 (1 VS/UWS, 1 MCS) patients had none. The average overlap rate was 52% (Figure 4 and Table 4).

**Table 4:** **Overlap between twice-scanned own name task**

		**Own name 1**				**Own name 2**			
**Patients**	**Left**		**Right**		**Left**		**Right**		**Overlap rate**
	**H**	**L**	**H**	**L**	**H**	**L**	**H**	**L**	
VS5	√	√	×	√	√	√	√	√	75%
VS6	×	√	√	√	√	√	√	√	75%
VS7	×	√	×	√	×	√	×	×	75%
VS11	√	√	×	×	×	×	√	√	0
VS21	×	√	×	√	×	×	×	√	75%
VS22	√	√	×	√	×	√	×	×	50%
VS23	√	√	√	√	×	√	×	×	25%
MCS5	√	√	×	√	√	√	√	√	75%
MCS7	√	√	√	√	×	×	√	√	50%
MCS8	√	√	√	√	√	√	×	×	50%
MCS9	√	√	√	√	×	×	×	×	0
MCS10	×	√	√	√	×	×	×	×	25%
MCS13	√	√	×	×	√	√	×	×	100%
								Average	52%

Overlap between twice-scanned own name task, right and left side auditory cortex was divided into primary (lower, L) and higher (H) order area.

## Discussion

In this study, using fMRI, we detected the cerebral responses of 66 DOC patients when hearing their own name spoken by a familiar voice (SON-FV). We correlated the activation patterns with the clinical outcome assessed with the CRS-R revised scale performed at 3, 6, and 9 months after scanning. It should be noted that ‘lower level’ and ‘higher level’ brain activation patterns refer to what is classically observed in DOC patients (see previous data from [3,6]) and that the current convenience sample of patients includes relatively more traumatic patients, hence biasing the obtained frequency of activation patterns to SON.

We found that BOLD signal in auditory cortex elicited by SON could statistically reliably predict the outcome in VS/UWS, particularly in traumatic patients. In VS/UWS patients, the overall predictive sensitivity and specificity was 67% and 81%. However, when taking into account brain injury etiology, the predictive value resulted higher for traumatic etiology. Specifically, 92% of traumatic VS/UWS patients with higher level activation extending to the higher order associate auditory cortex had a good outcome, whilst non-traumatic VS/UWS patients had a high negative predictive value, meaning that 85% patients with no activation or primary activation in auditory cortex achieved no recovery.

The reported data from the visual analysis of fMRI activation patterns (primary vs. higher order auditory activation) of low versus high were corroborated by ROC curve analysis, which supported the correlation between the activation of bilateral primary and secondary auditory cortex and VS/UWS patients’ recovery when the activation volume was >5.355 cm^3^ (all 39 VS/UWS patients) or >3.680 cm^3^ (traumatic VS/UWS patients). Patient age was also considered when assessing the prognostic value of the cerebral response elicited by SON-FV, and we could see that patients <40 years old had a better outcome, regardless of activation type. The average overlap rate was 52% between twice-scanned own name task.

The above findings emphasize the importance of VS/UWS patients’ etiology in the relationship between BOLD signals and outcome [20]. In particular, the two previously reported VS/UWS patients with higher level activation and good recovery were all traumatic [6].

The explanation as to why a higher level BOLD signal is associated with higher prognostic value in traumatic patients than in non-traumatic patients is still unknown and requires further study. One explanation could be the difference between the type of neuron injury sustained by traumatic and non-traumatic patients [21], particularly in the thalamus, which seems to play a key role in sustaining VS/UWS [22]. In fact, with non-traumatic lesions, the neurons have undergone ischemic necrosis and are subsequently no longer able to function. Traumatic lesions, however, are characterized by diffuse axonal injury, which does not involve neuron loss; if axon restoration is delayed after injury, it is conceivable to expect the neurons to work again since the neuron substrate is intact [21]. Given this, it is possible to speculate that the higher level auditory activation in traumatic VS/UWS patients, to a certain degree, may be taken as an initial sign of the recovery pattern related to the initial restoration of the axons, which may be a precursor to voluntary behavior reinstatement.

According to our behavioral assessment results, 4 out of 16 VS/UWS patients with higher level activation did not have a positive clinical outcome. Only 1 of these 4 patients, a 69 year old, had a traumatic etiology. The age of the patient may explain the lack of correlation between higher level activation and clinical outcome in traumatic patients. In fact, it has previously been shown that patients >40 years old have much lower chances of recovery, as confirmed herein. The other 3 patients were non-traumatic. Various complications may have affected the recovery process of these patients and the activation pattern could not reveal anything more about this population. In contrast, 3 VS/UWS patients with primary activation and 2 VS/UWS patients with no activation in the auditory cortex unexpectedly achieved good recovery according to follow-up behavior assessment. The arousal fluctuation or impairment often caused by the brain injury in VS/UWS [23] may partly explain the absence of activation. Furthermore, head movement in some VS/UWS patients, which occurred more frequently than in healthy subjects, may also have contributed to these false negative results to a certain extent. Finally, possible neurovascular coupling alterations or anatomical displacement of the auditory cortex in severely damaged brains might have caused altered or absent activation as measured by fMRI [24].

In this study, we did not find any statistically significant correlation between brain activation elicited by SON-FV or activation volume in auditory cortex and the clinical outcome of patients diagnosed as MCS or EMCS. Among these patients, 5 MCS and 1 EMCS had activation limited to the primary auditory cortex. Although previous studies report that MCS and EMCS patients may exhibit the same activation pattern to different kinds of stimuli similar to healthy controls, inconsistency along with issues related to head movement have shown to partially affect the BOLD signal [25-27].

In our opinion, there may be two main explanations of these results. Firstly, there’s the fluctuating level of consciousness typical of these patients [28]. For example, the patient may be asleep at the moment of scanning, making it, therefore, impossible to detect any brain activity in response to the stimuli. Secondly, the MCS is a very heterogeneous diagnostic category, which has recently been subcategorized into two groups, MCS+ and MCS–. The clear cut differentiation is based on the complexity level of observed behavioral responses and, more importantly, on the presence of command following, which has also been supported by neuroimaging findings [29]. In our study, these patients were not subcategorized. It could be that with better patient categorization, slight clinical improvements that could, to some extent, ameliorate the correlation between brain activation related to SON-FV and clinical outcome could be detected.

It should be emphasized that the employed contrast comparing the SON to noise does not permit strong cognitive interpretations (given that both stimuli differ for multiple semantic, emotional, and other physical parameters), and we cannot rule out the possibility to obtain similar results using other simpler stimuli than the patients’ names. The methodology employed herein was chosen from a clinical perspective and based on previous studies in order to increase the probability to obtain a cerebral response [6].

Finally, to overcome the limitation posed by arousal fluctuation in this challenging population of patients, we decided to test the consistency of the brain activation elicited by SON-FV in a subgroup of 13 patients (VS/UWS = 7, MCS = 6). We found that the mean overlap rate of this fMRI paradigm was nearly 52%, whilst some patients manifested no overlap. These results suggest that, similarly to clinical assessment administered to patients, repetitive SON-FV fMRI acquisition should be carried out in order to obtain more reliable prognostic information [25,30].

Some caveats could be pointed out when assessing the validity of our findings, such as the difficulty in accurately identifying primary and associative auditory cortex in severely damaged and grossly deformed brains and the uncertainties associated with intermodal (MRI-fMRI) image registration. For clinical purposes, an easy performing analysis method is important. ROC analysis could be an appropriate choice, because obtaining the activation volume was easier than accurately identifying activation patterns in the auditory cortex. From our ROC results, the total activation volume in bilateral auditory cortices could act as an index for predicting the recovery of VS/UWS patients.

It is also worth noting that we did not rigorously control the duration between scanning and brain injury. Brain injury severity or distribution was also not taken into consideration. The follow-up program could also have had some limitations, such as when patients were discharged from the hospital or transported long distance to another hospital and follow-up assessment by phone could have been partially affected by the families’ subjectivity.

The new data obtained by our study represents a substantial addition to a growing body of literature documenting the utility of brain imaging in this challenging population of patients [3,31,32]. However, before a consensus statement can be made regarding the use of fMRI for clinical purposes in the field of consciousness disorders, further study taking into account the utility of fMRI multi-scanning and the need to validate standardized paradigms that can be routinely used in clinical assessment is still needed.

## Conclusions

This large cohort study provides encouraging evidence suggesting that the simple and easily performed SON-FV fMRI paradigm could be considered a promising prognostic tool for VS/UWS patients in daily clinical use. In addition, we have demonstrated that the prognostic value of this method is higher in patients with traumatic rather than non-traumatic brain injury and that it is good practice to repeat this fMRI task in order to obtain more reliable prognostic information.

## Abbreviations

AFNI: Analysis of Functional NeuroImages
BOLD: Blood-oxygen-level-dependent
CRS-R: Coma Recovery Scale-Revised
DOC: Disorder of consciousness
EMCS: Emerging minimally conscious state
fMRI: Functional magnetic resonance imaging
FV: Familiar voice
HG: Heschl gyrus
MCS: Minimally conscious state
ROC: Receiver Operating Characteristic
SON: Subject’s own name
UWS: Unresponsive wakefulness syndrome
VS: Vegetative state
